# A Computed Tomography (CT)-Based Observational Study of Anatomical Variations in the Sphenoid Sinus: Implications for Surgical Planning and Patient Outcomes

**DOI:** 10.7759/cureus.66410

**Published:** 2024-08-07

**Authors:** Yash Jain, Vinod Shinde, Manu Babu

**Affiliations:** 1 Department of Otolaryngology, Head and Neck Surgery, Dr. D. Y. Patil Medical College, Hospital and Research Centre, Dr. D. Y. Patil Vidyapeeth, Pune, IND

**Keywords:** presellar, conchal pneumatization, optic nerve, onodi cell, internal carotid artery (ica), sphenoid sinus anatomy, conchal sphenoid sinus

## Abstract

Introduction: The sphenoid sinus (SS), a paired paranasal sinus located within the sphenoid bone, is crucial in various physiological and pathological processes. Its anatomical variations are of significant interest in clinical practice, particularly in otolaryngology, neurosurgery, and radiology. This study aims to determine the anatomical variations of the SS and related structures using computed tomography (CT).

Materials and methods: An observational study was conducted at a tertiary care center. The study included 300 patients aged 7-70 who underwent CT brain scans. Exclusions included prior sinonasal surgeries, tumors, nasal polyposis, recurrent pituitary lesions, head trauma, and past orbital or cranial surgeries. Three-dimensional reconstructions assessed SS dimensions, pneumatization types, and variations in the internal carotid artery and optic nerve.

Results: A study on the types of SSs revealed that the reseller type is the most common, accounting for 45% of cases (135 instances). The sellar type accounts for 36% (110 instances), while the conchal type is the least common, observed in 18.33% of cases (55 instances). A significant association between the SS type and variations between neurovascular structures was seen, which was confirmed using chi-square tests. There was a statistically significant relationship between carotid artery variations and SS, with the normal course being predominant at 200 individuals (73.33%). Approximately 40 cases (13.33%) present with dehiscence through the sinus, while 30 individuals (10%) show close proximity. Other, less common variations are observed in 10 patients (3.33%). Optic nerve variations displayed distinct frequencies, with the normal course prevailing in 250 cases (83.33%). Approximately 30 cases (10%) exhibit close proximity to surrounding structures, while 15 cases (5%) present with dehiscence through the sinus. Other less common optic nerve variations are observed in five patients (1.67%).

Conclusion: Comprehensive knowledge of SS anatomy through CT scans is essential for enhancing surgical outcomes and ensuring patient safety.

## Introduction

Enclosed beneath the sphenoid bone, the sphenoid sinus (SS) is the most inaccessible paranasal sinus and is closely associated with several important neuronal and vascular processes [1,2]. The ostium of the SS is located in the cranial region of the intranasal surface. The drainage channel from the SSs and posterior ethmoidal air cells into the superior nasal meatus is known as the sphenoethmoidal recess [3].

While the general anatomy of the SS is well established, considerable interindividual variations exist, making it a subject of immense interest and importance for medical professionals. The advent of advanced imaging techniques, particularly computed tomography (CT) scans, has revolutionized our ability to explore and understand the anatomical intricacies of the SS. Our study focuses on the anatomical variations within the SS, utilizing CT scans as a powerful tool for comprehensive analysis [4-6].

The SS, often referred to as the "key sinus" due to its central location, is a pneumatized cavity within the body of the sphenoid bone. Despite its relatively hidden position, the SS is of paramount importance in clinical practice. It is surrounded by vital structures, including the optic nerve, carotid artery, and pituitary gland, underscoring its anatomical and clinical relevance [7].

Understanding the normal and variant anatomy of the SS is crucial for various medical specialties, such as otolaryngology, neurosurgery, and radiology. Variations in SS anatomy may predispose individuals to certain complications during surgical procedures or contribute to the development of sinus-related pathologies. Hence, a comprehensive study focusing on anatomical variations becomes imperative for enhancing clinical knowledge and improving patient outcomes [8].

CT imaging stands as the cornerstone in the assessment of the SS. Its ability to produce high-resolution, cross-sectional images allows for a detailed examination of the sinus in three dimensions. With CT scans, clinicians can visualize the dimensions, configuration, and pneumatization of the SS with exceptional clarity. Additionally, contrast-enhanced CT scans facilitate the assessment of vascular structures and potential pathologies within the sinus. The precision offered by CT imaging makes it the primary choice for preoperative planning, particularly in cases of sinus surgeries or interventions involving adjacent structures [9].

Our study focuses on significantly contributing to understanding SS anatomy by employing CT scans as a powerful diagnostic tool. Through meticulous investigation of anatomical variations, we aim to enhance the knowledge base of healthcare professionals, ultimately improving clinical practices and patient outcomes.

## Materials and methods

This observational study gathered data from patients at a tertiary care center, Dr. D. Y. Patil Medical College, Hospital and Research Centre, with diverse sinonasal, neurological, and neurosurgical conditions between October 1, 2022, and March 31, 2024. The sample size of the study was 300.

**Figure 1:**
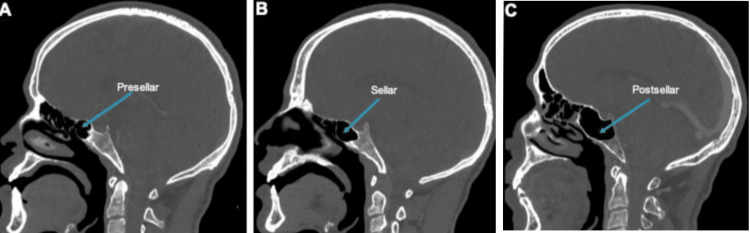
Sagittal section of a CT scan showing pneumatization of the SS. (A) Presellar pattern, (B) sellar pattern, and (C) conchal pattern of pneumatization (blue arrows) CT: computed tomography; SS: sphenoid sinus

The study included patients aged 7-70 undergoing CT brain scans, excluding those with SS pathologies or a history of sinonasal surgery, tumors, or nasal polyposis. Patients with primary pituitary lesions were included if they lacked SS pathologies and did not have recurrent pituitary lesions or a history of head trauma or related surgeries (Figure 1).

Institute Ethics Committee clearance was obtained with IESC/PGS/2022/120 before initiating the study. Participant informed consent was obtained from every participant before taking part in our study. A total of 300 CT scans of the SSs were analyzed from patients with a range of ENT, neurological, and neurosurgical conditions.

The scans were conducted using a bright-speed 64-slice CT scanner with a slice thickness of 3 mm, reconstructed to 0.5 mm, and windowed at 2,000-3,000 Hounsfield units. The study focused on measuring the maximum dimensions (length, height, and width) of each SS separately. Three-dimensional reconstructions were created using images from coronal, axial, and sagittal sections spaced 3 mm apart. Anatomical evaluations included identifying various pneumatization patterns, assessing the positions of the internal carotid artery (ICA) and optic nerve relative to the SS, and categorizing SS pneumatization into conchal, presellar, and sellar types. A descriptive statistical analysis was done using frequencies and percentages for categorical variables and mean and standard deviation for continuous variables. This was followed by an inferential statistical analysis, which was done using the chi-square test and independent samples t-test. Correlation analysis was done, and a p value of less than 0.05 was considered statistically significant for all tests.

## Results

The statistical analysis for the study utilized Statistical Package for the Social Sciences (SPSS) version 24 (IBM Corp., Armonk, NY). This analysis followed several structured steps. Initially, data from CT scans were entered into Microsoft Excel spreadsheets (Microsoft Corporation, Redmond, WA) and then imported into SPSS for further processing. Descriptive statistics were computed to summarize the dataset, encompassing frequencies and percentages for categorical variables such as types of pneumatization (conchal, presellar, and sellar) and the presence of anatomical variations like Onodi cells and variations in the ICA and optic nerve. These analyses provided insights into the frequency and characteristics of anatomical variations within the SS and their potential clinical implications.

The research includes individuals from Maharashtra, India, aged between 7 and 70, with a majority in the 31-40-year-old age group. Out of 300 patients, 60% are male, accounting for 180 individuals, while the remaining 40% are female, totaling 120 patients.

The study on the types of SSs revealed that the presellar type is the most common, accounting for 45% of cases (135 instances). The sellar type follows with 36% (110 instances), while the conchal type is the least common, observed in 18.33% of cases (55 instances). These findings highlight the variability in SS anatomy, which is crucial for surgical planning and intervention in the sphenoid region (Table 1).

**Table 1:** SS variations in patients SS: sphenoid sinus

SS type	Number of patients (frequency in %)
Conchal type	55 (18.33%)
Presellar type	135 (45%)
Sellar type	110 (36%)

**Figure 2:**
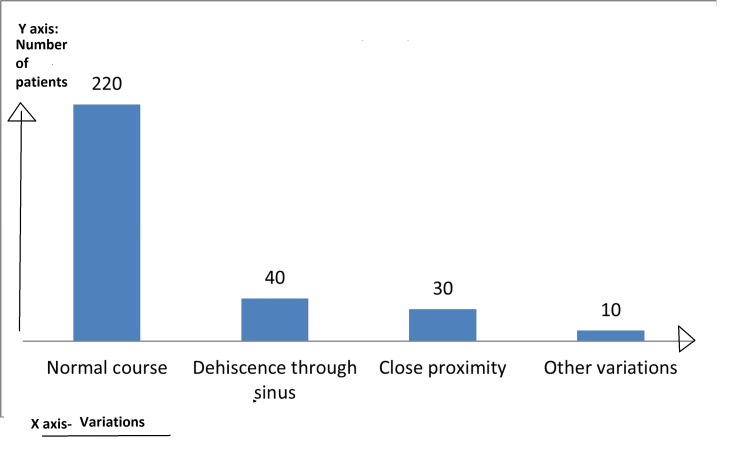
Variations of the ICA in relation to the SS The X-axis denotes anatomical variations of the ICA, and the Y-axis denotes the number of patients with variations ICA: internal carotid artery; SS: sphenoid sinus

The carotid artery variations within the patient cohort exhibit distinct frequencies, with the normal course being predominant at 200 individuals (73.33%). Approximately 40 cases (13.33%) present with dehiscence through the sinus, while 30 individuals (10%) show close proximity. Other, less common variations are observed in 10 patients (3.33%) (Figure 2).

The optic nerve variations among patients display distinct frequencies, with the normal course prevailing in 250 cases (83.33%). Approximately 30 cases (10%) exhibit close proximity to surrounding structures, while 15 cases (5%) present with dehiscence through the sinus. Other less common optic nerve variations are observed in five patients (1.67%) (Table 2).

**Table 2:** Variations of the optic nerve in relation to the SS SS: sphenoid sinus

Optic nerve variation	Number of patients (frequency in %)
Normal course	250 (83.33%)
Close proximity	30 (10%)
Dehiscence through sinus	15 (5%)
Other variations	5 (1.67%)

The study utilized the chi-square test to explore the relationship between anatomical variations of the SS and neurovascular structures, revealing statistically significant associations. In particular, the association between SS type and carotid artery variation yielded a chi-square value with 4 degrees of freedom (df) and a p value of <0.001, confirming a significant statistical relationship. Similarly, the association between SS type and optic nerve variation showed significance, with a chi-square value of 4 df and a p value of 0.027. However, the relationship between SS type and other neurovascular structures was deemed insignificant with a p value of 0.102, which might relate to a single-center study and a relatively smaller sample size, thereby necessitating multicenter studies in the future with a larger sample size. These findings underscore the importance of understanding SS anatomical variations in relation to adjacent neurovascular structures.

## Discussion

The SS, a paired paranasal sinus located within the sphenoid bone, plays a crucial role in various physiological and pathological processes. Its anatomical variations are of significant interest in clinical practice, particularly in otolaryngology, neurosurgery, and radiology [10-12].

These variations can affect surgical approaches to the base of the skull, the SS itself, and surrounding structures, thereby influencing the risk of complications. Due to its high-resolution imaging capabilities, CT scans are the preferred modality for visualizing these anatomical nuances [13,14]. Variations in the dimensions, configuration, and septation of the SS can affect its drainage patterns, the proximity of vital neurovascular structures such as the optic nerves and the carotid arteries, and even the spread of infections or tumors. Understanding these variations is essential for preoperative planning, reducing intraoperative risks, and enhancing diagnostic accuracy.

In our study, the presellar type is the most common, accounting for 45% of cases (135 instances). The sellar type accounts for 36% of cases (110 instances), while the conchal type is the least common, observed in 18.33% of cases (55 instances). These findings highlight the variability in SS anatomy, which is crucial for surgical planning and intervention in the sphenoid region. Carotid artery variations within the patient cohort exhibit distinct frequencies, with the normal course being predominant at 73.33%. Approximately 13.33% of cases present with dehiscence through the sinus, while 10% show close proximity. Other less common variations are observed in 3.33% of patients. Optic nerve variations among patients display distinct frequencies, with the normal course prevailing in 83.33% of cases. Approximately 10% exhibit close proximity to surrounding structures, while 5% present with dehiscence through the sinus. Other less common optic nerve variations are observed in 1.67% of patients.

Rahmati et al. used cone-beam computed tomography (CBCT) to study SS variations and adjacent structures in 103 patients (206 sides) [14]. They focused on pneumatization levels in the SS, anterior clinoid and pterygoid processes, and the protrusion of the optic canal, vidian canal, and foramen rotundum, along with sinus septa prevalence. Their findings highlighted significant associations: pneumatization of the pterygoid process correlated strongly with the vidian canal (p < 0.001) and foramen rotundum (p < 0.001) protrusions. In contrast, optic canal protrusion correlated significantly with pneumatization of the anterior clinoid and pterygoid processes (p < 0.001). Additionally, carotid canal protrusion was significantly related to pterygoid process pneumatization (p < 0.001). These complex anatomical variations underscored potential complications and symptoms, stressing the importance of detailed CBCT assessments for understanding SS anatomy in planning safe and effective paranasal surgeries [14].

Furthermore, Cho et al. studied neurovascular protrusion within the SS and its relationship with sinus air space. They examined 100 cadaveric heads, measuring the pneumatization and distances of structures like the ICA, optic nerve, maxillary nerve, and vidian nerve from the sinus walls. Results showed optic nerve, ICA segments 1 and 3, maxillary nerve, and vidian nerve bulging in varying percentages of cases, correlating with increased sinus pneumatization. Thin sinus walls were noted in fully pneumatized sella turcica, particularly affecting optic nerve proximity. The study concluded that greater pneumatization heightens the likelihood of neurovascular structures protruding into the sinus cavity [5].

In contrast, Baldea and Sandu emphasized the critical role of understanding SS anatomy, especially its pneumatization patterns, in skull base surgeries. Their retrospective analysis of 50 craniofacial CT scans focused on various SS types, with sellar and hypersinus types being the most common. They highlighted how hyperpneumatization can complicate endoscopic procedures by altering anatomical relationships with vital nerves and blood vessels. The study also explored correlations between pneumatization types, patient demographics, and sinus location. Overall, the research underscored the variability in SS pneumatization and its implications for surgical planning and risk assessment [15].

On the other hand, Famurewa et al. conducted a study on SS anatomical variations and their implications for the endoscopic endonasal transsphenoidal approach (EETA) in Nigerian adults. Analyzing CT scans of 320 patients, they categorized SS pneumatization into conchal (1.9%), presellar (1.2%), sellar (56.6%), and postsellar (40.2%) types. They observed lateral extensions of the SS into adjacent structures: pterygoid in 45.1%, greater wing in 35%, lesser wing in 11.6%, and full lateral type in 30.3% of cases. Intersphenoid septum (ISS) configuration included single (46.9%), multiple (50.6%), and absent (2.5%) types, with 31.6% showing ISS insertion into the ICA bony covering and 34.4% demonstrating ICA protrusion into the SS cavity. These findings underscore the need for detailed preoperative imaging to navigate these variations safely during EETA procedures in this demographic [16].

Overall, the current study adds to the existing literature by providing further evidence of the various anatomical variations of the SS and its clinical importance in surgeries like functional endoscopic sinus surgery, pituitary surgeries, clival surgeries, and other anterior skull base surgeries. Understanding the anatomical variations of the SS is crucial for clinicians and surgeons in fields like otolaryngology, neurosurgery, and radiology. Due to its proximity to vital structures such as the optic nerve, ICAs, and pituitary gland, the SS poses significant risks during surgical procedures. This study, utilizing CT scans, aims to outline these variations comprehensively. By mapping diverse anatomical configurations, the research seeks to improve preoperative planning, reduce intraoperative complications, and enhance surgical outcomes. Detailed knowledge of SS anatomy is essential for navigating procedures like endoscopic sinus surgeries and transsphenoidal hypophysectomies. Additionally, understanding the relationships between the SS and adjacent neurovascular structures can enhance diagnostic accuracy and guide more effective therapeutic strategies. Ultimately, this research aims to provide personalized surgical approaches, minimizing risks and improving patient care by precisely understanding individual anatomy.

The study's limitations include a potentially inadequate sample size for capturing all SS anatomical variations comprehensively across different demographics and conditions and possible selection bias due to exclusion criteria, limiting the generalizability of findings to broader patient groups. Future research should aim to mitigate these biases to enhance understanding of SS anatomy and variations. A larger multicentric study, including the implications of the variations in various surgical techniques, would benefit clinicians and surgeons in understanding and treating various SS pathologies better.

## Conclusions

This study highlights the significant anatomical variations of the SS as observed through CT scans, underscoring the complexity and individual differences in sinus anatomy. Understanding these variations is vital for enhancing the safety and effectiveness of surgical interventions near the SS. The findings emphasize the importance of detailed preoperative imaging and individualized surgical planning to minimize risks and optimize patient outcomes. By providing a comprehensive map of SS variations, this research contributes to improved diagnostic accuracy and surgical precision, ultimately advancing patient care in otolaryngology and related fields. While we attempted to perform a comprehensive analysis of anatomical variations of SS, there is much scope for further research. A larger multicentric study including the implications of the variations in various surgical techniques would be beneficial for clinicians and surgeons to better understand and treat various SS pathologies.
